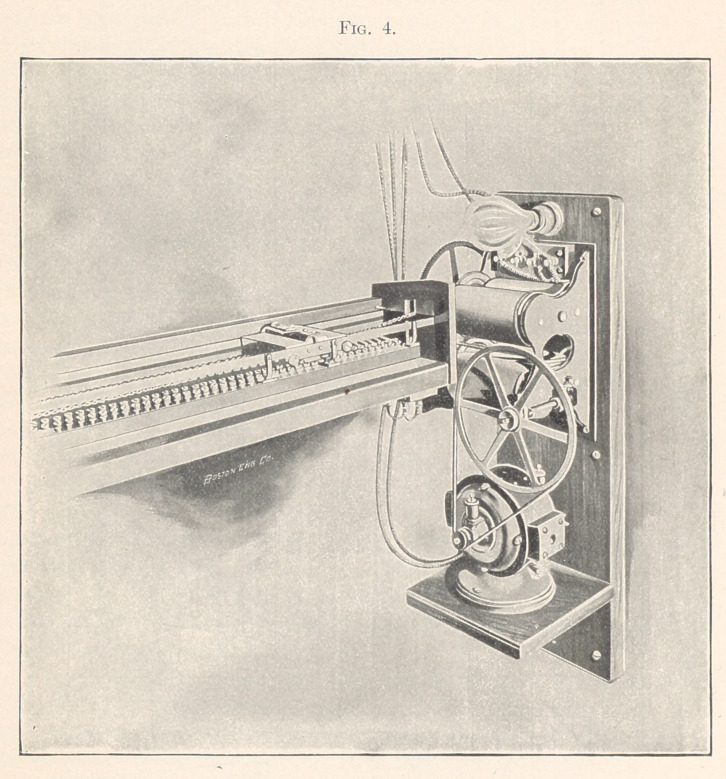# Dental Notes

**Published:** 1903-03

**Authors:** William Rollins

**Affiliations:** Boston, Mass.


### DENTAL NOTES.

BY WILLIAM ROLLINS, BOSTON, MASS.

### NOTE VII. ANÆSTHESIA OF THE DENTINE BY ELECTRICITY.

That anæsthesia of the dentine could be produced by driving cocaine into it by electricity was proved by McGraw before 1889. Since that time it has been used regularly in my office. In 1896 I had occasion to call attention to these facts in this journal, as the method was being claimed by others. In this paper I mentioned principles on which rheostats for using the street current should be constructed, if the method was to be used with safety and comfort. The principles were discovered as the result of a long experience in making rheostats for dental purposes. I endeavored to show that the ordinary method of placing the resistance on one wire was not safe, as it made it possible for the patient to be exposed to a heavy current. I therefore divided the resistance equally between the two wires. I also stated that in addition to the variable resistance there should be a fixed resistance on each wire, that the maximum current which could reach the variable resistance could be only four mialliampères, when the current was used for dentine anæsthesia.

I also found that to produce least pain the current should be as far as possible a regularly increasing one. As it was difficult at that time to produce such a rheostat in a compact form, which would permit the current to vary from one-one-hundredth of a milliampère to two milliampères, which was about the average range required in dentine work, I advocated making the current increase by imperceptible steps, describing revolving rheostats containing two hundred steps that I had designed, and which had been made and offered for sale by Clark & Mills. The instruments had met with little favor, as the importance of a minute division was not understood and they cost more than those with fewer steps on a shunt plan. A more perfect rheostat was designed the same year, and made for me by the same firm in 1897. Since this instrument was placed in my office it has worked well. A number of dentists interested in the subject have been to see it, and approved of the multistep principle, though several, among whom I mention Dr. Price (who saw it in 1900), did not approve of placing the patient in the direct circuit, preferring the ordinary shunt plant.

I have delayed describing the instrument partly on this account and partly because of investigations on X-light. Now the instrument has been well tested, and I publish this brief description, though I have since invented another rheostat in which there are no steps, the legions of electrons advancing steadily in ever-increasing columns. This later rheostat contains a principle new in medical rheostats, the current increasing steadily through the diminished resistance of selenium when exposed to light. There are countless ways of embodying this principle in a rheostat. I mention it here, as it should be interesting to men who are working on medical rheostats. Later, when time and inclination serve, I shall show drawings of such rheostats.

### DESCRIPTION OF MULTISTEP RHEOSTAT.

This rheostat is divided into three separate parts,—first, a maximum current resistance placed high up on the wall; second, a variable multistep resistance; third, a fixed resistance within a few inches of the patient. All three parts are divided into halves, and one-half of the resistance in each is placed on each wire of the 110-volt direct street current. All the resistances are carbon rods made especially for this rheostat by the Dixon Company. The carbon rods in the first and third parts are entirely enclosed in hard-rubber cells. The first and third rheostats have each a fixed resistance of about fifty thousand ohms. The second, or variable multistep resistance contains fourteen hundred and fifty-two carbon rods. The minimum current through all three parts is one-one-hundredth of a milliampère; the maximum, two milliampères. The smallest resistance in the variable part is ninety ohms; the highest is about two million ohms.

These carbons were all separately tested before use and so arranged that each gives an equal increase of current at each step. The resistance of the patient, even if high, need not be taken very seriously, as it is a negligible quantity in comparison. Even if it were possible for the patient to offer a resistance of a hundred thousand ohms, the current would still be over a milliampère. With a low-voltage instrument of ten volts, a similar resistance would mean a current not far from a tenth of a milliampère.

No figures are given of the first and third, or fixed resistances, as their construction should be clear from the text. Four figures are given of the second, or multistep variable resistance, as this is quite complicated. It is contained in a wooden case with glass front, the whole about fifteen and a half feet long. Inside the case are blocks of hard rubber collectively fifteen feet long. These contain four rows of carbon cylinders arranged in pairs, the two rows on one side being connected with one wire of the street main after it has passed through the wall resistance, the two rows on the other side with the other wire also similarly passed through the wall resistance. The maximum current brought to this rheostat, then, is only that which will pass through a resistance of fifty thousand ohms. The tops of the carbon rods are inserted in metal caps with polished tops projecting from the rubber and connected with the moving carriage shown in Fig. 4 by springs. This carriage controls the amount of current that can get through the rheostat. When it is at one end the maximum current is one-one-hundredth of a milliampère. When it is at the other, the current is two milliampères. The movements of the carriage are controlled by a switch within reach of the patient and dentist. By means of this switch the carriage can be moved forward or back either fast or slow. The slow speed cuts out resistance so slowly that it requires twenty minutes of continuous movement of the carriage to increase the current to the maximum. I find that this is as fast as is usually desirable. As the position and speed of the carriage are under the control of the patient and the operator, the operation can be conducted at the speed which they consider best. The carriage is moved by an endless chain which is controlled by two magnetic clutches, shown in Fig. 4, through the foot or chair switch.

Figs. 1, 2, and 3 are so clear they need no particular description, though it may be well to mention that the carriage is held against the two side-rails by springs to insure perfect contact with the carbons. Though the drawings have been made with great care to scale, the principal dimensions are also marked in feet. Any one in want of such a rheostat could probably have it most cheaply made by the Clark & Mills Electrical Company, of Boston, as they have had experience in making me a number of rheostats. The calculation of the required resistance of each carbon is a matter taking time and the testing of a very great number of carbons

**Fig. 1**

**Fig. 2**

**Fig. 3**

Fig. 4.

is also a long task. One would at first think that each carbon should have the same resistance as all the others, to make the current increase regularly and gradually, but further thought will show that the matter is not of that order of simplicity. Before closing this paper I desire to again call attention to a method of making dentine insensible by the use of high-voltage currents of minute ampèrage (I spoke of this matter in the paper published in this journal in 1899, and already alluded to in the present paper), and to the method of relieving the pain of an alveolar abscess and stopping its development described in my paper in this journal in 1897. I expect to see these methods reinvented about this time.

## Figures and Tables

**Fig.1 f1:**
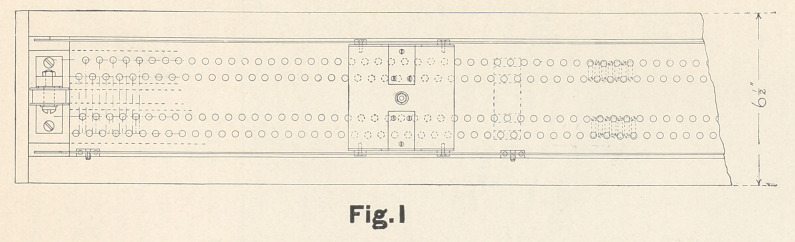


**Fig.2 f2:**
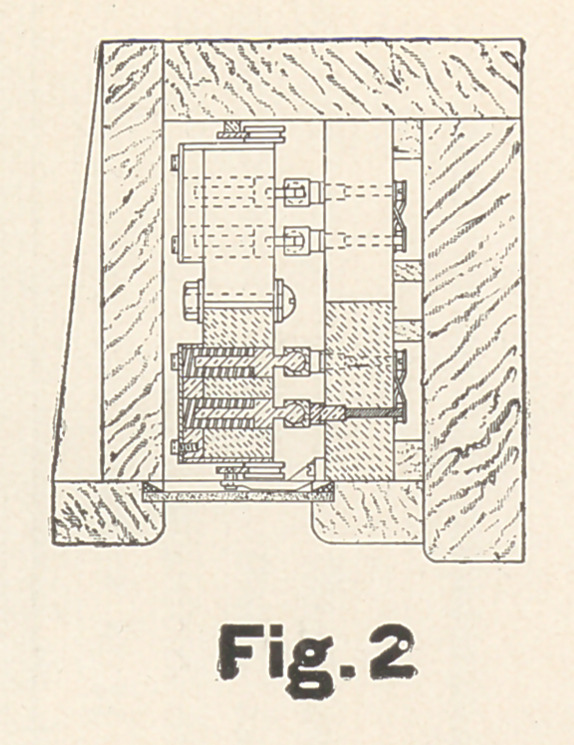


**Fig.3 f3:**
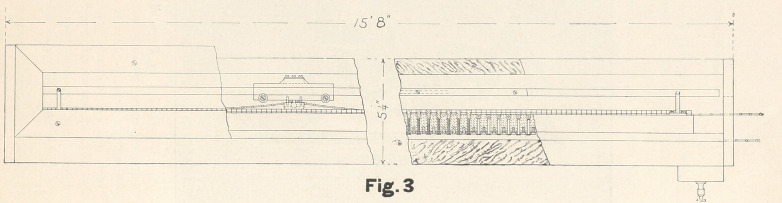


**Fig. 4. f4:**